# Quantitative Assessment and Spatial Analysis of Metals and Metalloids in Soil Using the Geo-Accumulation Index in the Capital Town of Romblon Province, Philippines

**DOI:** 10.3390/toxics10110633

**Published:** 2022-10-22

**Authors:** Delia B. Senoro, Cris Edward F. Monjardin, Eddie G. Fetalvero, Zidrick Ed C. Benjamin, Alejandro Felipe B. Gorospe, Kevin Lawrence M. de Jesus, Mark Lawrence G. Ical, Jonathan P. Wong

**Affiliations:** 1 Resiliency and Sustainable Development Center, Yuchengco Innovation Center, Mapua University, 658 Muralla St., Intramuros, Manila 1002, Philippines; 2 School of Civil, Environmental and Geological Engineering, Mapua University, 658 Muralla St., Intramuros, Manila 1002, Philippines; 3 School of Graduate Studies, Mapua University, 658 Muralla St., Intramuros, Manila 1002, Philippines; 4 Mapua-RSU Joint Research Laboratory, Romblon State University, Sawang, Romblon 5500, Philippines; 5 Research and Development Office, Romblon State University, Odiongan, Romblon 5505, Philippines; 6 Electrical Engineering Department, Romblon State University, Odiongan, Romblon 5505, Philippines

**Keywords:** geo-accumulation, metals and metalloids, sediments, spatial analysis, soils

## Abstract

The municipality of Romblon in the Philippines is an island known for its marble industry. The subsurface of the Philippines is known for its limestone. The production of marble into slab, tiles, and novelty items requires heavy equipment to cut rocks and boulders. The finishing of marble requires polishing to smoothen the surface. During the manufacturing process, massive amounts of particulates and slurry are produced, and with a lack of technology and human expertise, the environment can be adversely affected. Hence, this study assessed and monitored the environmental conditions in the municipality of Romblon, particularly the soils and sediments, which were affected due to uncontrolled discharges and particulates deposition. A total of fifty-six soil and twenty-three sediment samples were collected and used to estimate the metal and metalloid (MM) concentrations in the whole area using a neural network-particle swarm optimization inverse distance weighting model (NN-PSO). There were nine MMs; e.g., As, Cr, Ni, Pb, Cu, Ba, Mn, Zn and Fe, with significant concentrations detected in the area in both soils and sediments. The geo-accumulation index was computed to assess the level of contamination in the area, and only the soil exhibited contamination with zinc, while others were still on a safe level. Nemerow’s pollution index (NPI) was calculated for the samples collected, and soil was evaluated and seen to have a light pollution level, while sediment was considered as “clean”. Furthermore, the single ecological risk (Er) index for both soil and sediment samples was considered to be a low pollution risk because all values of Er were less than 40.

## 1. Introduction

Metal and metalloid (MM) contamination in soils and sediments, and its subsequent release into groundwater or surface waters, is considered to be a major environmental concern. The extent of MM contamination can be attributed to many factors, such as microbial activity, the physicochemical properties of the media, and location sources [1]. Globally, rapid industrialization with uncontrolled discharges are major sources of high concentrations of metalloids such as antimony (Sb), arsenic (As), and boron (B), and metals such as cadmium (Cd), chromium (Cr), copper, (Cu), lead (Pb), molybdenum (Mo), vanadium (Va), and zinc (Zn). Industrial inputs to water bodies may come from various sources, which include industrial effluents, sewage wastewater, fossil energy combustion, land deposition from landfills and industrial emissions [1,2,3,4], and agricultural wastes.

Soil pollution and its adverse effects by heavy metal contamination are often irreversible [5], and the detection of its point sources can be a serious challenge. The MMs are mostly inorganic, and normally As, Cd, Cr, Cu, Pb, mercury (Hg), nickel (Ni), and Zn have affinity to soils. The specific environmental media (i.e., soil and sediments) are the major sink of MMs. This creates a potential hazard and health risks to humans through skin contact and accidental ingestion of contaminated soil, in addition to the risk of ingestion of these MMs through groundwater (GW) and soil–plant–human or soil–plant–animal–human interaction in the food chain. The presence of elevated MMs can negatively affect vegetation by reducing farm produce and food quality, consequently affecting food and nutrition security [5]. On the other hand, sediments are produced by forces breaking rocks and turning them into small particles that are carried by river flow [6]. According to Monjardin et al. [7], land use change also affects the water availability in certain areas, which is related to the river flow that produces these sediments. Usually, sediments tend to accumulate in areas where the river slope is at a minimum and the river does not produce enough hydraulic energy to carry these sedimentary particles.

Elevated MM concentration in soil can be due to natural causes or anthropogenic activities. The spatial heterogeneity of MMs in soils can be a result of topography, climate, or other natural factors [8] including typhoons [9], which transport the soils and sediments. On the other hand, anthropogenic activities can cause MMs to accumulate in the soil, especially when there are mine tailings and uncontrolled dumpsites. Petrochemical products such as leaded gasoline, lead-based paints, fertilizers, and pesticides, along with coal combustion residues, also contribute to contamination when the accumulated amount exceeds the allowable limits [10]. Thus, it is important to monitor the concentration of MMs, and to determine the possible sources of contamination in order to effectively implement programs and prevent and/or reduce soil pollution by MMs.

Romblon town, the capital municipality of the province of Romblon in the Philippines, is part of the Romblon Island Group located at the center of the archipelago and is almost wholly underlain by the Romblon metamorphic rocks [11]. It is a third-class municipality, with a land area of 86.87 km^2^ and a population of more than 40,000 people, where the primary livelihood is agriculture, livestock raising, and marble mining and processing. Romblon is known as the marble capital of the Philippines, having the largest and highest-quality marble deposits. The increasing population and the aspiration to develop more marble-based products raised the concern of the community due to environmental pollution, particularly soil contamination by MMs, which has been associated with mining activities. The mining of marble in Romblon for commercial use started in the 1970s. However, to date, there has been no research conducted in the area to determine the presence and extent of MM accumulation in soils in Romblon. Hence, this research was carried out in order to provide information to the community and local government units to allow them to create appropriate programs and necessary interventions.

According to research by Adepoju and Adekoya [12], even small- to medium-sized mining operations can contaminate sediments with various heavy metals such as Ag, As, Cd, Co, Cr, Cu, Hg, Ni, Pb, and Zn. Additionally, it was also found that the sediments receiving marble mine discharge showed elevated concentrations of potentially harmful elements such Cu, Zn, Pb, As, Cr, Hg, Ni, and Co [13]. Furthermore, significant Cd concentrations in soils in the marble mining district were recorded in 2019 [14]. These findings were associated with marble mining activities. There were also significant concentrations of Zn in the study area that were linked to emissions from the trucks and other machinery used in marble quarrying. The abovementioned recorded studies explicitly described the human health risks when there are marble mining activities in the area.

Spatial concentration maps have been frequently used in the monitoring of the quality of soils and sediments impacted by heavy metals. In the Asian continent, the use of spatial concentration mapping in soils and sediments has been broadly implemented and includes research carried out in the western Asian region [15,16], the central Asian region [17,18], the south Asian region [19,20], the east Asian region [21,22], and the southeast Asian region [23,24]. Actual sampling can be used to assess the quality of soils and sediments, but it is time consuming and labor intensive. Moreover, the density of the datapoints has an impact on the precision of the maps created using these sampling data [25].

The employment of innovative tools, such as machine learning methods, is consistent with the interdisciplinary shift toward Industry 4.0 and has become increasingly common over the past few years. One of the focal points of Industry 4.0 is artificial intelligence. This is crucial for the development of various technical specialties [26]. The Artificial Neural Network (ANN) is an effective system that is primarily based on biological neural network systems. In this instance, an ANN is made up of artificial neurons [27]. ANNs have been broadly implemented in spatial estimation in soils and sediments, including in the study of soil erosion [28], soil salinity [29], soil metal pollution [30], rainfall-runoff sediment process [31], and suspended sediment concentration estimation [32]. However, there are some limitations and drawbacks in the use of ANN, such as having trouble in crossing plateaus of the error function landscape. To address this issue, optimization algorithms such as Particle Swarm Optimization (PSO), which utilizes a global search feature, could further improve the ANN model performance [33]. The PSO effectively uses probability-based transition procedures to conduct concurrent searches of the solution hyperspace without formally assuming derivative information. The physical model, that underpins the transition rules, assumes emergent group action results from the social interaction of bird flocks and fish schools [34]. Therefore, this new research is focused on determining the concentration and distribution of some MMs in Romblon’s soils. This has been carried out to evaluate the quality of the soil and the potential ecological risk posed by various MMs, and to calculate the geo-accumulation index that determines the level of MM accumulation in soils.

## 2. Materials and Methods

### 2.1. Study Area and Sampling Points

**Figure 1:**
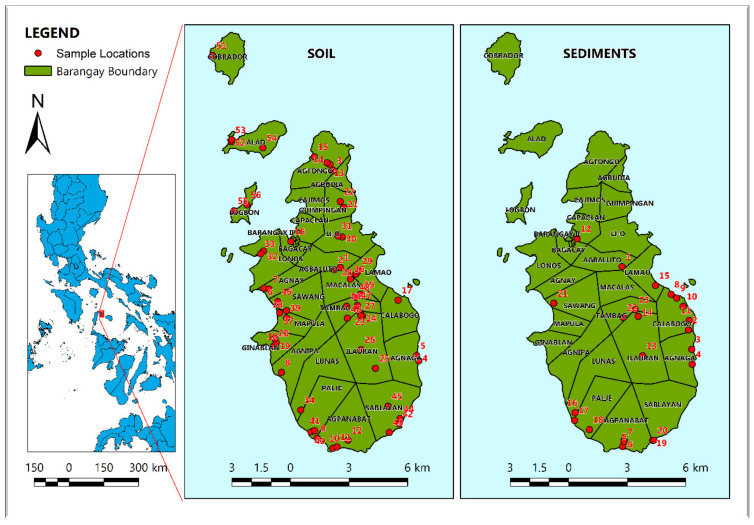
Study Area and Sampling Locations in Romblon island. The numbers at the bottom of the figure represent the map scale.

The island municipality of Romblon is located in the Philippines, with coordinates of 12.5294° N and 122.2881° E. The climate of the island falls under Type III of the Corona climatic classification system, with no pronounced wet and dry seasons, but it is relatively dry from January to May. Its topography is mostly composed of hills and it has a maximum elevation of approximately 400 m above sea level. The soil and sediment samples collected were spatially distributed across the municipality of Romblon, as shown in Figure 1.

### 2.2. Collection of Samples

A total of fifty-six soil and twenty-three sediment samples were collected from twenty-six barangays of the municipality of Romblon. The sampling locations were spatially distributed over the island municipality of Romblon, targeting barangays that would best represent the whole area. Top soils were collected using a grab stainless trowel, while sediment was collected using an Eckman [35] grab sampler. Twenty-five soil and twelve sediment samples were collected from agricultural and residential areas. Coordinates were recorded using a handheld Garmin global positioning system (GPS), model Montana 680. Collected samples were transported to the RSU-Mapua joint research laboratory in Sawang, Romblon Municipality, for laboratory preparation and analysis.

### 2.3. Sample Preparation and Analysis

The preparation and MM concentration analysis were conducted at the Mapua-RSU Joint Laboratory at RSU Sawang campus in the municipality of Romblon, Philippines. The soil and sediment samples were stored in zipper bags upon collection and transported to the laboratory for preparation following the work of Wang et al. [36] and EPA No. LSASDPROC-300-R4 [37]. The acquired soil samples were dried at 68 °C for 3 h, grounded into a fine powder using an agate mortar and pestle, homogenized, and passed through a 10-mesh sieve before analysis. The concentration of nine metals, As, Ba, Cr, Cu, Fe, Mn, Ni, Pb, and Zn, was analyzed using a handheld portable Olympus Vanta X-Ray Fluorescence analyzer (pXRF). This portable device is known to perform both in-situ and in the laboratory for the detection of MMs, with an accuracy comparable to ICP [23,38]. The pXRF was calibrated using the Olympus Vanta blank and set to Geochem mode before analysis. The data curation, validation, modeling, and visualization were carried out in the Yuchengco Innovation Center of Mapua University, Manila, Philippines.

### 2.4. Assessment of Metal Pollution in Soils and Sediments

The contamination levels of the soil were assessed using Single Factor index (*P_i_*) and Nemerow’s pollution index (NPI) values (*P_n_*) [38]. The computations of the two indices, *P_i_* and *P_n_*, were calculated based on Equations (1) and (2).

(1) $$P_{i}=\sqrt{\frac{C_{i}}{S_{i}}}$$

where in *P_i_* is the single factor index, *C_i_* is the heavy metal concentrations in the soil samples (mg/kg), and *S_i_* is the soil quality standard value for each element (mg/kg) [39].

(2) $$P_{n}=\sqrt{\frac{{\left(P_{i_{\max}}\right)}^{2}+{\left(P_{i_{ave}}\right)}^{2}}{2}}$$

The Nemerow’s synthetic pollution index can be calculated using the equation above. Wherein, *Pi*_max_ is the maximum value of the single factor index and *Pi_ave_* is the average value of the computed *P_i_*. The grading standards for the pollution indices are shown in Table 1 [38,39].

The potential ecological risk index (*RI*) developed by Hackanson was widely used to assess the ecological dangers of heavy metal pollution in sediments, soils, and water [2]. The computation of *RI* is shown by Equation (3)

(3) $$RI=\overset{n}{\underset{i=1}{\sum }}Er_{i}=\overset{n}{\underset{i=1}{\sum }}P_{i}\times T_{i}\;$$

where *RI* represents the total number of risk factors observed in the soil samples, *E_r_* indicates the monomial potential ecological risk factor for each component, and *T_i_* is the metal toxic response factor. The *T_i_* values for Pb, Cr, Ni, Mn, Cu, and Cd were 5, 2, 5, 1, 5, and 30, respectively [4]. The grading standards for the risk index are shown in Table 1.

### 2.5. Correlation Analysis

Pearson’s correlation was performed using OriginLab 2021 to look at how a certain metal concentration affects the other concentrations of various metals found in soils and sediments. This is also used to identify the groupings of MMs.

### 2.6. Spatial Analysis

ArcGIS was used to render the spatial distribution map for the concentration of metals by spatial interpolation. Spatial interpolation is the process of using points with known values to estimate values at other unknown points. It is a method of creating surface data from sample points. One of the simplest and most popular interpolation techniques is inverse distance weighted (IDW) interpolation, which enforces the estimated value of a point that was influenced more by nearby known points rather than those farther away. In IDW interpolation, the sample points are weighted during interpolation, such that the influence of one point relative to another declines with the distance from the unknown point to be determined. Conventional deterministic approaches, on the other hand, were frequently created under certain constraints and do not work in different real-world situations. The performance is restricted using default settings and the scarcity of accurate observations. Due to the ANN-PSO algorithm’s capacity for modeling non-linear datasets and its adaptation to real-world circumstances, combined with the IDW approach, provides solution to this concern [40].

### 2.7. Geo-Accumulation Index

Another way to identify the degree of contamination of soils and sediments would be by using Muller’s geo-accumulation index. This method compares the background concentration of heavy metals in the area versus its current concentration [41]. Background concentration is the level of concentration that is normal in a certain area. This can be found in places that are free from economic and human activity. Equation 4 shows the formula to calculate the geo-accumulation index, where its values correspond to seven levels of contamination.

(4) $$I_{geo}=\log_{2}\left(\frac{C_{n}}{1.5B_{n}}\right)$$

where *C_n_* is the measured concentration of the element in the environment and *B_n_* is the geo-chemical background value in the soil.

The following values indicate the degree of contamination in soil: *I_geo_ of* ≤ 0, no contamination; 0 < *I_geo_* ≤ 1, no contamination to moderately contaminated; 1 < *I_geo_* ≤ 2, moderately contaminated; 2 < *I_geo_* ≤ 3, moderately to heavily contaminated; 3 < *I_geo_* ≤ 4, heavily contaminated; 4 < *I_geo_* ≤ 5, heavily to extremely contaminated; and *I_geo_* ≥ 5, extremely contaminated [42,43].

## 3. Results and Discussion

### 3.1. Concentration of Metals and Metalloids in Soil Samples

The MMs concentration in soil and sediment samples were recorded and showed that the highest concentration detected in soil was 41.25 mg/kg (Ba), 236.60 mg/kg (Pb), 10.60 mg/kg (Cr), 31.28 mg/kg (Ni), 591.84 mg/kg (Mn), 233.10 mg/kg (Cu), 0.005 mg/kg (As), 6367.01 mg/kg (Fe), and 542.96 mg/kg (Zn). While sediments showed maximum concentrations of 13.71 mg/kg (Ba), 37.59 mg/kg (Pb), 12.91 mg/kg (Cr), 13.71 mg/kg (Ni), 14.93 mg/kg (Mn), 233.10 mg/kg (Cu), 0.003 mg/kg (As), 3201.14 mg/kg (Fe), and 13.422 mg/kg (Zn). Table 2 shows the comparison between measured MMs concentration and the corresponding allowable concentrations based on soil quality standards (SQS) and sediment quality guidelines (SGV). It was recorded that the maximum values of three heavy metals (Pb, Mn, Cu) exceeded the SQS value. This is perceived to be dangerous and should be subjected for mitigation. The average concentration for each MMs was observed to be within the limits of the SQS. Standard deviations are also shown in the table. The SDs have higher values compared to mean concentration which indicated that there were high variations between datasets. There were many areas with low metal concentrations, while there were also small areas who have much higher concentrations. The results showed that the Romblon municipality in totality is still within the acceptable MMs concentration range, although there are specific areas that exceeded the allowable values, which should be mitigated. It can be perceived that there were no imminent dangers and health risks posed by MMs concentrations in soils and sediments in the municipality of Romblon. However, the local government shall make strategic plans concerning how to maintain these metal concentrations within allowable levels. In addition, there was no Cd concentration recorded in soils and sediments. According to the findings of Usman et al. [38], soil acidity and plant absorption prevented the detection of Cd in the soil. It was explicitly stated that Cd accumulates in the shoots and is remedied by plants through phytoextraction [35,43]. 

### 3.2. Assessment of Metal Pollution in Soils

Nemerow’s pollution index was determined in order to assess the degree of soil contamination in the municipality of Romblon. The *P_n_* value shows how much pollution each metal is likely to contribute to contaminate a certain environmental medium.

**Figure 2:**
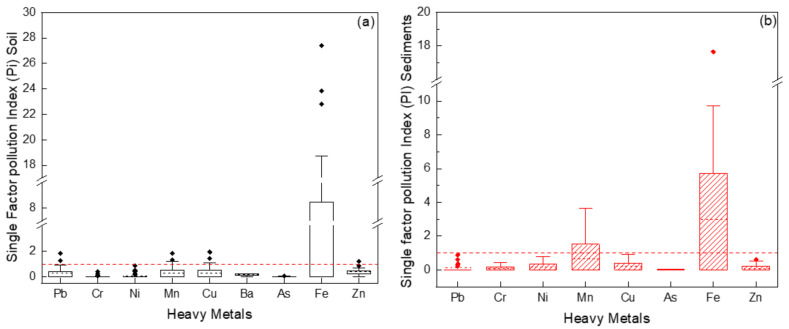
(**a**) Box plot of single factor pollution index (P_i_) of Pb, Cr, Ni, Mn, Cu, Ba, As, Fe, and Zn in Romblon Municipality for soil; (**b**) Box plot of single factor pollution index (P_i_) of Pb, Cr, Ni, Mn, Cu, Ba, As, Fe, Zn in Romblon Municipality for sediments.

**Figure 3:**
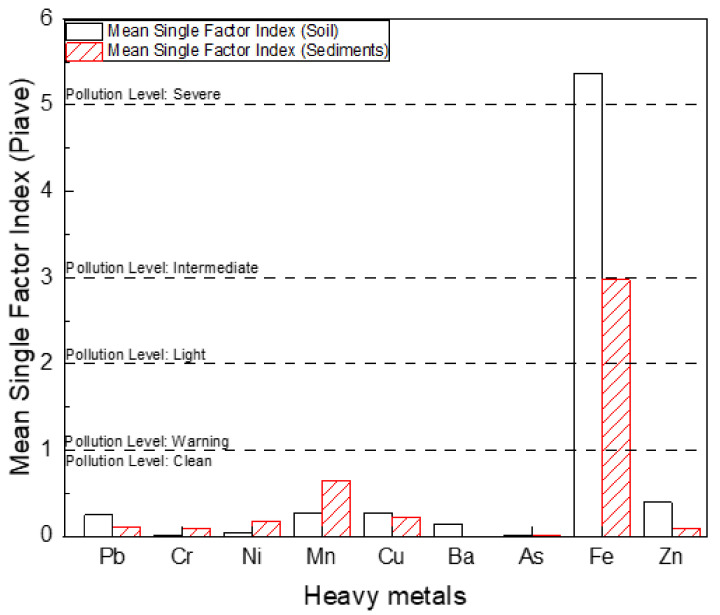
Mean Single Factor pollution index of Pb, Cr, Ni, Mn, Cu, Ba, As, Fe, and Zn for soil and sediment.

As shown in Table 3, the Romblon municipality has a *P_n_* value of 1.72 for soil, indicating a light pollution level contamination across the municipality. In comparison to the single factor pollution indices, the values of *P_n_* were higher due to the few samples that exhibited a higher MMs concentration, because *P_n_* accounted for the highest index observed in these sets of data. This was illustrated by the outliers in Figure 2a. The sediments have a *P_n_* value that also indicated a clean pollution level for the municipality. Because no observed *P_i_* values reached intermediate or severe level pollution, this minimized the risk of metal contamination that may pose a threat upon human exposure through dermal contact, incidental ingestion, and impact by agricultural crops quality [36,37,47,48,49]. The mean *P_i_* values of soil and sediments from the municipality were observed to be less than 1, as shown in Figure 3. This implied that the soil and sediments across the municipality is at Class I, with a clean pollution level (Table 1). This showed that there was no evidence of MMs contamination throughout the municipality.

**Figure 4:**
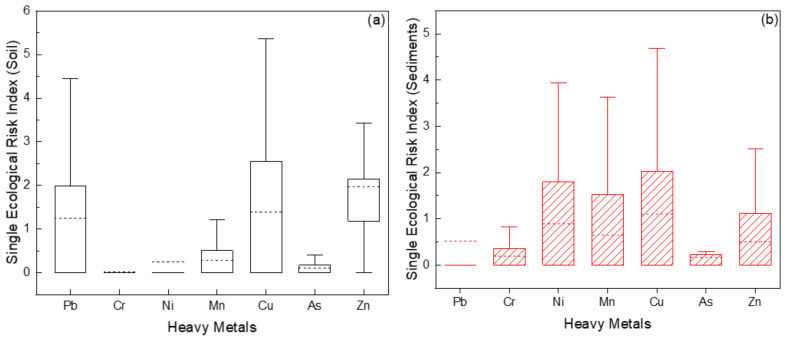
(**a**) Boxplot of single ecological risk index of Pb, Cr, Ni, Mn, Cu, As, and Zn for soil; (**b**) Boxplot of single ecological risk index of Pb, Cr, Ni, Mn, Cu, As, and Zn for sediments.

The study area’s single ecological risk index for soil and sediments had an order of Cd < Cr < Ni < Mn < Pb < Cu and Cd < Pb < Mn < Cr < Ni < Cu, respectively, as shown in Figure 4a,b. In general, for all MMs found in soils and sediments, most of the sites had low potential ecological risk index values. Furthermore, no sampling points in the study area exceeded the single potential ecological risk index (Er) value of 40. According to grading standards, this *E_r_* value indicated that there was low pollution risk in soil and that the soil was clean.

### 3.3. Correlation Analysis

**Figure 5:**
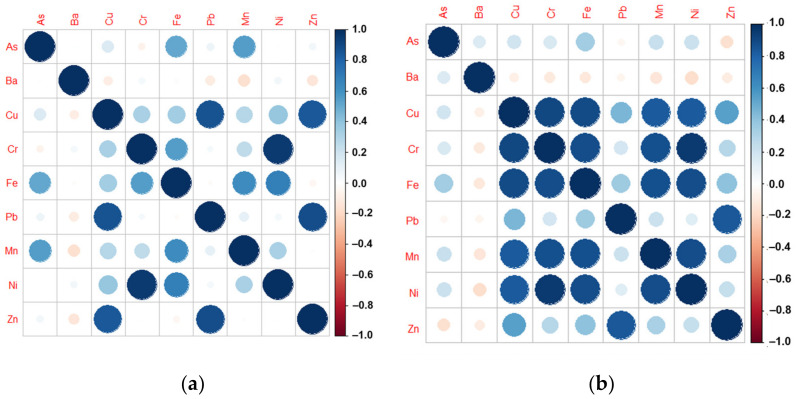
Correlation matrix for (**a**) Soils; (**b**) Sediments.

Pearson correlation analysis was used to determine the relationship between MMs in both soils and sediments. The degree of metal interdependence and association in both soils and sediments was determined. The correlation coefficients and matrices of Ni-Cr, Ni-Mn, Ni-Cu, and Cu-Pb were 0.957, 0.423, 0.376, and 0.886 (*p* < 0.01), respectively, for soils as shown in Figure 5a. The correlation coefficients of Cr-Ni, Cr-Mn, Cr-Cu, Ni-Mn, Ni-Cu, and Cu-Mn were 0.9521, 0.8703, 0.9045, 0.8880, 0.8383, and 0.8345, respectively, for sediments, as shown in Figure 5b. The value at *p* < 0.01 expresses the statistical significance of the relationship and association of metals in soil and sediments. The correlation matrix for sediments and soils are presented in Figure 5a,b.

### 3.4. Spatial Analysis using NN-PSO-Inverse Distance Weighted Interpolation

The spatial distributions of the MMs of both soils and sediments in the municipality of Romblon using NN-PSO with IDW interpolation technique are shown in Appendix A. It was found out that there was elevated concentration of up to 25.06−41.22 mg/kg of Ba in soil on the main island of Romblon. Meanwhile, the highest concentrations of up to 5.26−10.55 mg/kg of Cr and 13.08–31.14 mg/kg of Ni in soil were found in the southeastern areas of the main island. On the other hand, concentrations of up to 142.59–233.07 mg/kg of Cu, 139.16–236.56 mg/kg of Pb, and 255.23–591.64 mg/kg of Mn in soil were found in both the southeastern areas of the main island and Logbon island. Elevated concentrations of Mn in soil were also found in the northeast part of the main island. Lastly, Cd in soil was not detected in Romblon.

**Figure 6:**
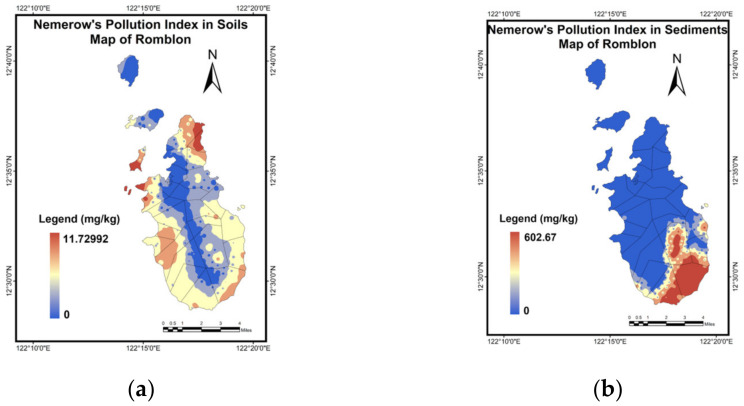
NN-PSO-IDW map for (**a**) Soils; (**b**) Sediments.

The highest concentrations of Ba in sediments of up to 6.89–13.71 mg/kg were found in the central part of the main island. For Cr, Cu, Pb, and Ni, the highest concentrations in sediments were in the central-south to southern part of the main island, with values of up to 3.77–12.80 mg/kg, 14.85–43.50 mg/kg, 20.35–37.58 mg/kg, and 4.31–14.80 mg/kg, respectively. Meanwhile, the highest concentrations of Mn in sediments of up to 118.50–392.41 mg/kg are in the central-south to southern and western portions of the main island. Lastly, Cd in sediments was not detected in Romblon as well. Considering the overall effect of the group of metals detected in the area of study, Figure 6a,b exhibited the spatial map of the NPI for soils and sediments.

The results suggest that there was movement of metals from the mountains or higher elevations and accumulation of the same in the lower elevations, which might be due to erosion or weathering effects. This can be supported by studies conducted to identify sources of Pb contamination and accumulation. It suggests that complex topography is more conducive to soil Pb accumulation, as it often affects soil movement due to runoff erosion and topographic differences that allow the enrichment of soil with heavy metals [8]. The concentration distribution of Cr, Cu, Pb, and Ni in soil and sediments appeared to be very similar. Combinations of metals in either medium that can be compared are Cr and Ni in soil, Cu and Pb in soil, and Cr, Cu, Pb, and Ni in sediments.

Cr and Ni are mostly found in ultramafic rocks, typically ranging from <LOD to 100 mg/kg [50]. Another study also concluded that Cr and Ni are highly correlated and similar in spatial distributions in contaminated soils [10]. In a study conducted by Huang et al. [10], a high correlation and similar spatial distribution between these three elements, Cr, Cu, and Ni, existed when there is the presence of soil pollution due to anthropogenic activities. Another study also identified that metals such as Cr, Cu, and Pb exist in roadside soils, and their concentration decreases with the distance from the road and is due to traffic emissions [51] and the wearing of tires and brake linings from vehicles [3].

The NN-PSO simulation results are presented in Table 4 and Table 5 for soils and sediments, respectively. This includes the structure of the models. The training algorithm used was the Levenberg–Marquardt algorithm because it is the fastest method for moderately sized networks [52]. The transfer function employed was the hyperbolic tangent sigmoid function, as suggested by De Jesus et al. [53], while the number of iterations used in the simulations was 2000 [54]. The regression plots for validation and testing are exhibited in Appendix B.

### 3.5. Geo-Accumulation Index

**Figure 7:**
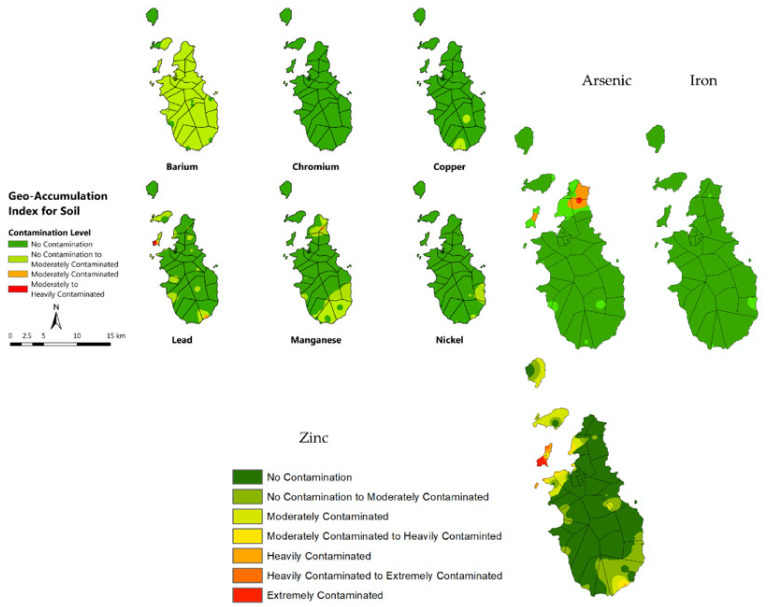
Geo-accumulation index of soil for Ba, Cr, Cu, Pb, Mn, Ni, As, Fe, and Zn.

**Figure 8:**
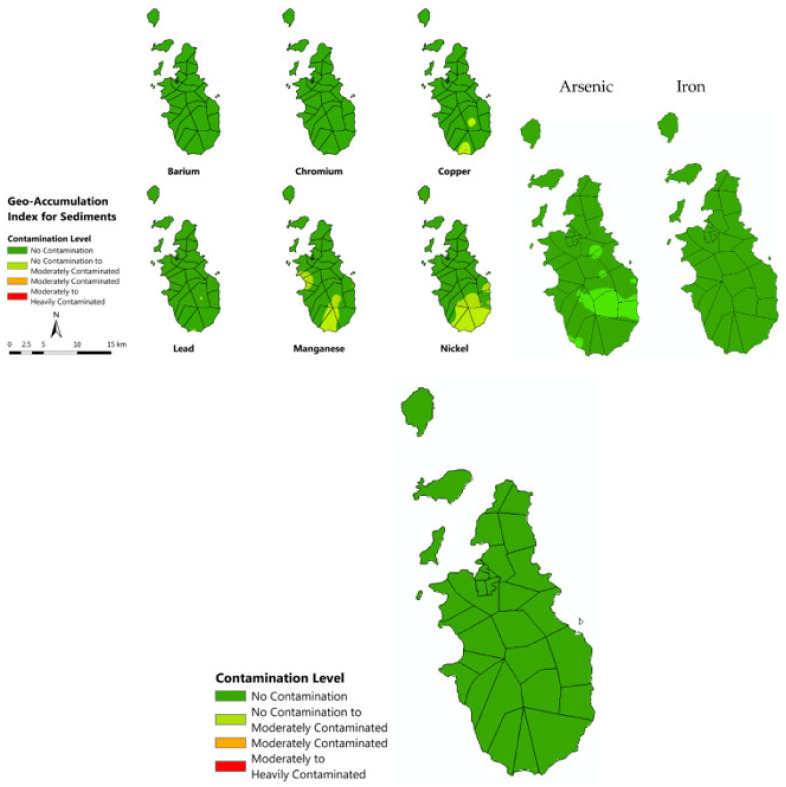
Geo-accumulation index of sediments for Ba, Cr, Cu, Pb, Mn, Ni, As, Fe, and Zn.

Figure 7 and Figure 8 show the computed geo-accumulation index of the following MMs: Ba, Cr, Cu, Pb, Mn, Ni, As, Fe, and Zn. The geo-accumulation index has already been used by many researchers to assess the level of heavy metal and metalloid elements in soil [55,56]. This is being achieved by comparing the measured concentration with its background concentration. Considering the geo-accumulation index for all MMs in soil, Zn showed the highest accumulation, followed by Ba, Mn, As, Pb, Ni, Cu, Cr, and Fe. Almost all the areas in Romblon Municipality recorded “no contamination to moderately contaminated” with respect to Ba. This means that Ba accumulation in the area is happening; however not in an alarming level. The Cr, on the other hand, showed no evidence of contamination in the whole municipality of Romblon. An alarming level of Pb was recorded at Logbon Island, where the geo-accumulation index was within the range of “moderately to heavily contaminated”. This should be given special attention by the local government unit (LGU) because exposures to elevated concentrations of Pb could cause serious health problems, such as high blood pressure, joint and muscle pain, headache, and mood disorders [57]. Also, the geo-accumulation index tells us that these elements were increasing in concentration at a very fast pace. The Zn registered the highest geo-accumulation index among all the metals presented. Logbon island was categorized as being extremely contaminated. This is alarming, and LGU should be aware and create appropriate intervention. There is a significant possibility that the community may be exposed to elevated Zn concentrations which might cause severe iron deficiency as Zn inhibits the uptake of Fe. Also, As poses a threat to people living in the northern part of the island, as the results showed that there is a buildup of As concentration in the area. This is something that the LGU has to give attention to in order to ensure the safety of inhabitants. The northern area is the only place on the island that is moderately contaminated with As. Sediments, on the other hand, did not exhibit alarming sediments geo-accumulation level as it moved from upstream to downstream. Sediments do not stay in one place for a long period of time, which is why metal accumulation is not that significant [58]. The Ni and Mn showed the highest geo-accumulation index among all the metals measured but only at a level of “no contamination to moderately contaminated”. All other metals exhibit “no contamination” on the island. Below maps (Figure 7) could guide LGUs to plan an action on how to prevent continuous contamination in the environment. Many of these elements are still within the safe threshold; however, there is a significant possibility that it will reach its toxic level if not given proper intervention.

## 4. Discussion

The results showed that Romblon Municipality has low pollution risk level; however, current condition presented clear signs of being classified as contaminated in the near future as a result of human activities [59] if no intervention will be carried out. The study focused on determining the current soil and sediment heavy metal concentration level in the island using an XRF scanner. This technology has been very effective in accurately determining metal concentration levels in soil with convenience [60,61]. Nine MMs were detected that have significant concentration in the area, and these are listed as follows: Ba, Cr, Cu, Pb, Mn, Ni, As, Fe, and Zn. In this study, Nemerow’s pollution index was calculated to assess the potential of certain metal that contributed to the contamination of an environmental medium. Many studies have already made use of NPI to assess metals in soil contamination [62,63,64] and all produced a critical assessment as to how these metals contaminated the environment and at which level. In this study, NPI was calculated and resulted in a Pi value of less than 1. This result implied that the municipality was under the Class I category of having a “clean” level. It is fortunate that the area is still under this category; however, if no proper regulation is carried out, the environmental condition could become worse [65]. Another parameter considered in the study is the geo-accumulation index computation; this uses spatial analysis [66] to determine the index and locate it in a spatial map. The *I_geo_* of the nine MMs detected in the soils and sediments were computed. The Ni, Cr, Fe, and Cu in soil showed the lowest value of *I_geo_*, which means that these metals are not significantly accumulating in the municipality [67]. On the other hand, Mn, Pb, Ba, and Zn produced higher value of *I_geo_* in some parts of the municipality, especially in the northern area, giving the idea that something is happening in the area that caused the significant accumulation of metal. The Mn reached a moderately contaminated *I_geo_* level in the northern part of Romblon, while Pb and As reached the heavily contaminated *I_geo_* level in that region. Logbon Island was found to be extremely contaminated with Zn. Too much uptake of Zn could produce a severe deficiency in Fe. No significant contamination level was found for the sediment samples collected around the whole island. Only the Mn and Ni were elevated, but only at the “no contamination to moderately contaminated” level and covered only a small area south of the island. Fortunately, all other metal concentrations were still on the “no contamination” level. The *I_geo_* is a useful tool to assess the current contamination level in a certain area for sediments [68,69] and soils [70,71]. It gives accurate assessment that could aid in planning certain mitigation actions. The maps produced in this study presented hotspot locations that should be given attention, and the local government shall create a plan on how this could be mitigated and managed. The Ba is another metal of great concern in the area.The *I_geo_* level is on “no contamination to moderately contaminated” and covered almost the entire municipality. This level indicates that possible significant metal accumulation might happen in the area soon. The Ba is a dangerous metal because when ingested could cause hypokalemia, hypertension, cardiac arrhythmia, and skeletal muscle paralysis [72].

## 5. Conclusions

In this study, the contamination levels of MMs in the soils and sediments across the municipality of Romblon in the Philippines were assessed and monitored. Soil and sediment samples were collected from the 26 barangays. All samples were analyzed for the presence of As, Ba, Pb, Cr, Cu, Mn, Ni, Zn, and Fe and assessed for potential ecological risk by both soil and sediments. It was recorded that all mean metal concentrations in the soils and sediments were within the SQS and SGV, except for the Mn and Fe in sediments. The highest metal concentration recorded from fifty-six soil samples were as follows: Fe (3258.36 mg/kg), Mn (49.44 mg/kg), and Zn (109.58 mg/kg); and for twenty-three sediment samples, Fe (1146.44 mg/kg), Mn (41.09 mg/kg), and Zn (6.08 mg/kg). An X-ray fluorescence scanner was used to measure this concentration with comparable accuracy to ICP. Another index used to assess this metal concentration was the geo-accumulation index, which identifies the contamination level in a certain area. The *I_geo_* exhibits levels of contamination and gives us an idea of which metals tend to accumulate in the area at a significant rate. In soil, while Fe has the highest concentration, Zn was the one to exhibit the highest geo-accumulation index that reached up to the “heavily contaminated” level. The Fe showed no contamination because its level does not change significantly and it is perceived as being naturally occurring in the area. No alarming *I_geo_* level was computed for sediments, and the highest level was only at the “no contamination to moderately contaminated” level and was found only for the metals Ni and Mn. Logbon island is an area that should be closely monitored as it shows an alarming level of heavy contamination from Zn and Ba. The *P_n_* values of the soil and sediment samples from the municipality were under Class II and Class I, respectively. This indicated a light pollution level for soil and a clean for sediments. Furthermore, the single ecological risk index of the heavy metals from both soil and sediment samples were considered as “low pollution risk” because the values of all the soils and sediments are Er < 40. The calculated mean pERIs for both soils and sediments were considerably low, which supports the *P_i_* and *P_n_* for no pollutants across the municipality.

## Figures and Tables

**Table 1:** Grading standards for pollution indices [2].

Class of Pollution	I	II	III	IV	V
*P_i_*	≤1.0	1.0–2.0	2.0–3.0	3.0–5.0	>5.0
*P_n_*	≤0.7	0.7–1.0	1.0–2.0	2.0–3.0	>3.0
Pollution Level	Clean	Warning	Light	Intermediate	Severe
*E_r_*	<40	40 ≤ *E_r_* < 80	80 ≤ *E_r_* < 160	160 ≤ *E_r_* < 320	*E_r_* ≥320
Risk Index	<150	150–300	300–600	600–1200	>1200
Pollution Risk	Low	Moderate	Considerable	High	Very high

**Table 2:** Mean concentrations of MMs in soil (mg/kg) compared to soil quality standards (SQS) and sediment quality guidelines (SGV).

Metals	Media	Mean ± Std. Dev Concentration (mg/kg)	Range: Min–Max (mg/kg)	SQS/SGV (mg/kg)
Pb	Soil	12.951 ± 35.463	0–236.594	70 [44]
	Sediments	2.9803 ± 8.5015	0–37.5898	48 [45]
Cr	Soil	0.2481 ± 1.4537	0–10.5977	64 [44]
	Sediments	1.5720 ± 2.8782	0–12.9132	76 [45]
Ni	Soil	1.1217 ± 4.5621	0–31.2878	45 [44]
	Sediments	1.8447 ± 3.2672	0–14.933	24 [45]
Mn	Soil	49.436 ± 108.16	0–591.835	180 [44]
	Sediments	41.091 ± 88.314	0–395.884	30 [46]
Cu	Soil	14.470 ± 36.412	0–233.098	63 [44]
	Sediments	6.1405 ± 10.595	0–43.8773	50 [45]
Ba	Soil	18.724 ± 11.489	0–41.2469	750 [44]
	Sediments	1.0393 ± 3.1308	0–13.7099	-
As	Soil	0.0028 ± 0.0048	0–0.02743	12 [44]
	Sediments	0.0038 ± 0.0029	0–0.00914	11 [45]
Fe	Soil	3258.3645 ± 6367.0062	0–30,529.3	-
	Sediments	1446.4403 ± 3201.1433	0–12,682.3	15 [46]
Zn	Soil	109.5809 ± 542.9625	0–4080.59	250 [44]
	Sediments	6.0795 ± 13.4192	0–52.3562	140 [45]

**Table 3:** Single Factor Index, NPI, and Pollution Risk Index for Soil and Sediments.

Metals	Media	*Pi*_max_	*Pi_ave_*	Mean *P_n_* (Soil)	Mean *P_n_* (Sediments)	Mean *RI* (Soil)	Mean *RI* (Sediments)
Pb	Soil	1.8385	0.2490	19.37	12.49	3.19	3.98
	Sediments	0.8849	0.1036				
Cr	Soil	0.4069	0.0133				
	Sediments	0.4122	0.0932				
Ni	Soil	0.8338	0.0494				
	Sediments	0.7888	0.1771				
Mn	Soil	1.8133	0.2735				
	Sediments	3.6326	0.6489				
Cu	Soil	1.9235	0.2785				
	Sediments	0.9368	0.2190				
Ba	Soil	0.2345	0.1447				
	Sediments	-	-				
As	Soil	0.0478	0.0110				
	Sediments	0.0288	0.0154				
Fe	Soil	27.388	5.3682				
	Sediments	17.652	2.9776				
Zn	Soil	4.0401	0.3955				
	Sediments	0.6115	0.1002				

**Table 4:** NN-PSO Simulation Results for Soil Samples.

	Hidden Neurons	No. of Particles	No. of Iterations	Elapsed Time (Sec)	R Validation	R Testing
As	24	2	2000	127.71542	0.99075	0.99469
Ba	25	8	2000	129.91471	0.98974	0.98092
Cu	26	6	2000	125.81433	0.99723	0.97607
Cr	30	4	2000	136.83323	0.99912	0.99909
Fe	29	7	2000	125.62094	0.96847	0.99916
Pb	28	8	2000	125.53996	0.98120	0.99644
Mn	23	5	2000	125.44344	0.99711	0.99988
Ni	27	8	2000	124.70619	0.99992	0.99652
Zn	26	10	2000	125.64613	0.97132	0.99644
P_n_	30	1	2000	125.29881	0.95174	0.98768

**Table 5:** NN-PSO Simulation Results for Sediment Samples.

	Hidden Neurons	No. of Particles	No. of Iterations	Elapsed Time (Sec)	R Validation	R Testing
As	25	10	2000	133.36397	0.99121	0.99997
Ba	29	5	2000	140.33520	0.99996	0.98946
Cu	22	7	2000	127.38098	0.99398	0.99999
Cr	27	3	2000	132.87273	0.99994	0.99981
Fe	26	5	2000	132.74669	0.99710	0.99661
Pb	25	3	2000	134.09987	0.95636	0.99115
Mn	29	4	2000	223.43633	0.99842	0.99990
Ni	30	5	2000	204.67335	0.96586	0.98279
Zn	28	10	2000	200.37023	0.99905	0.99975
P_n_	29	6	2000	126.24143	0.99743	0.99441

**Figure A1:**
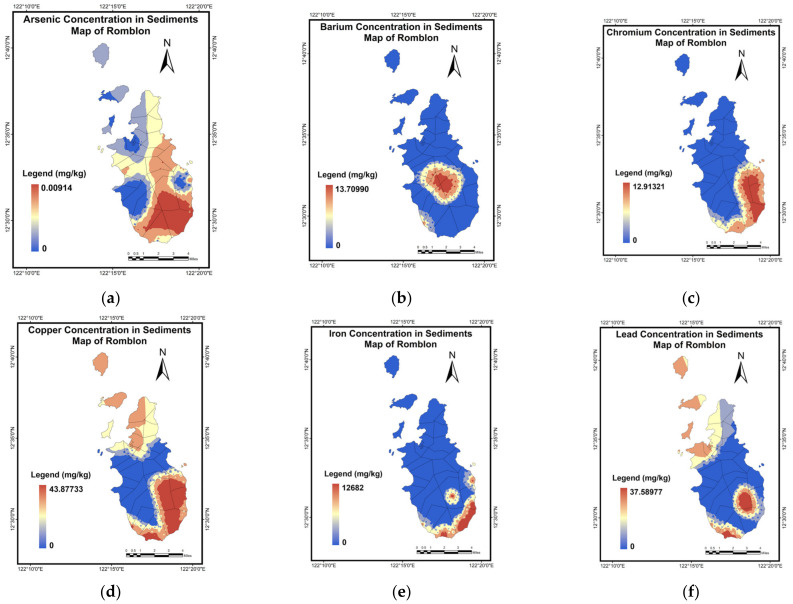

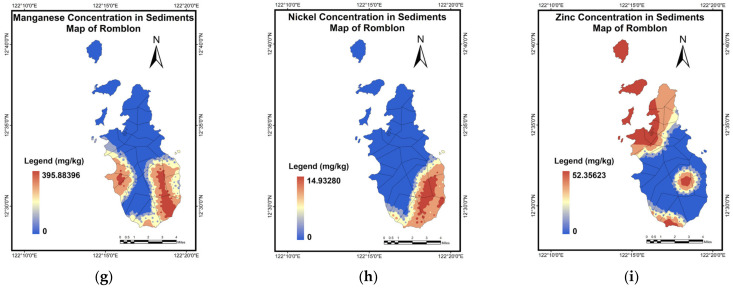
Heavy metal concentration of sediments: (**a**) Arsenic, (**b**) Barium, (**c**) Chromium, (**d**) Copper, (**e**) Iron, (**f**) Lead, (**g**) Manganese, (**h**) Nickel, (**i**) Zinc.

**Figure A2:**
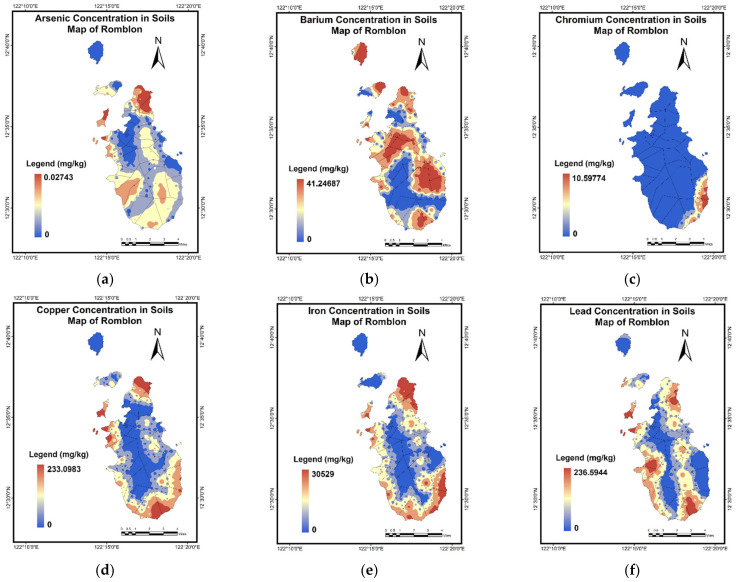

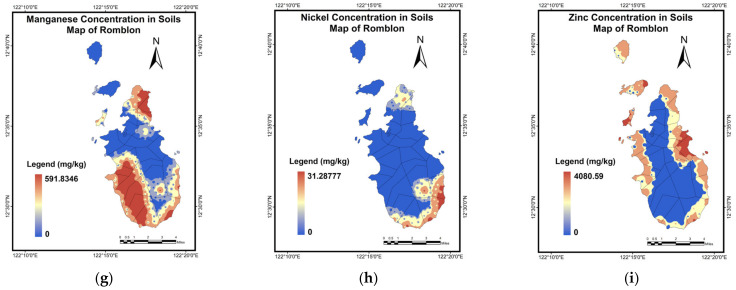
Heavy metal concentration of soils: (**a**) Arsenic, (**b**) Barium, (**c**) Chromium, (**d**) Copper, (**e**) Iron, (**f**) Lead, (**g**) Manganese, (**h**) Nickel, (**i**) Zinc.

**Figure A3:**
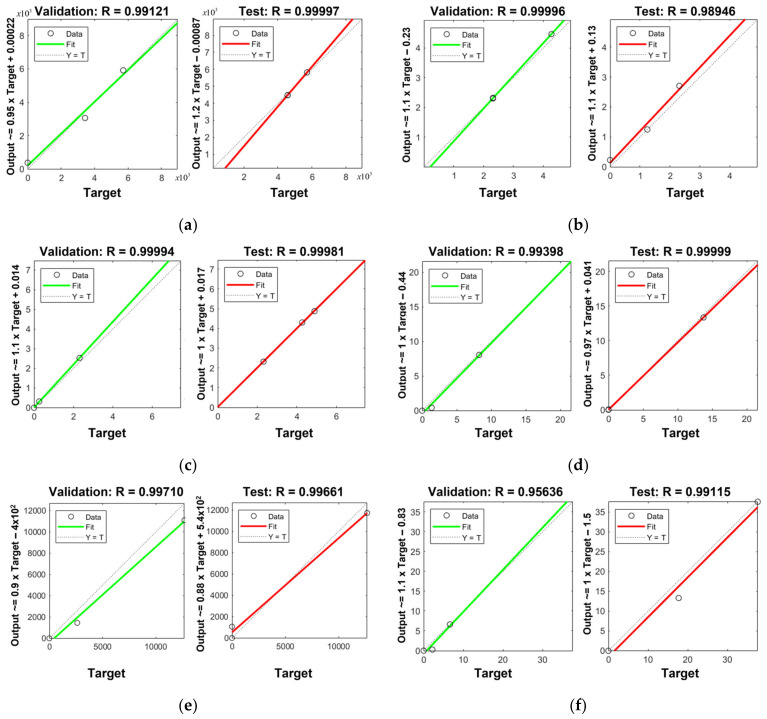

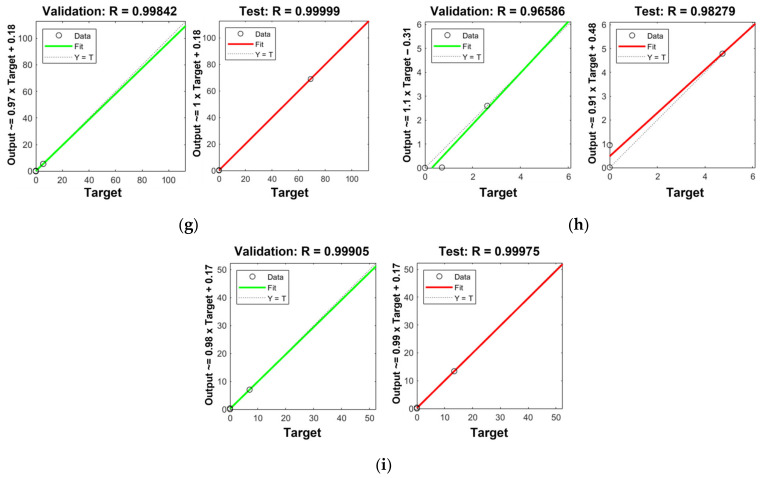
Regression plots for sediments: (**a**) Arsenic, (**b**) Barium, (**c**) Chromium, (**d**) Copper, (**e**) Iron, (**f**) Lead, (**g**) Manganese, (**h**) Nickel, (**i**) Zinc.

**Figure A4:**
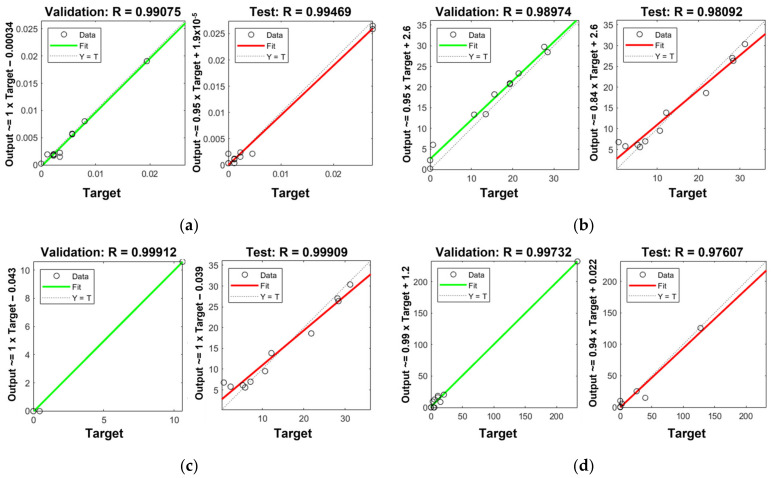

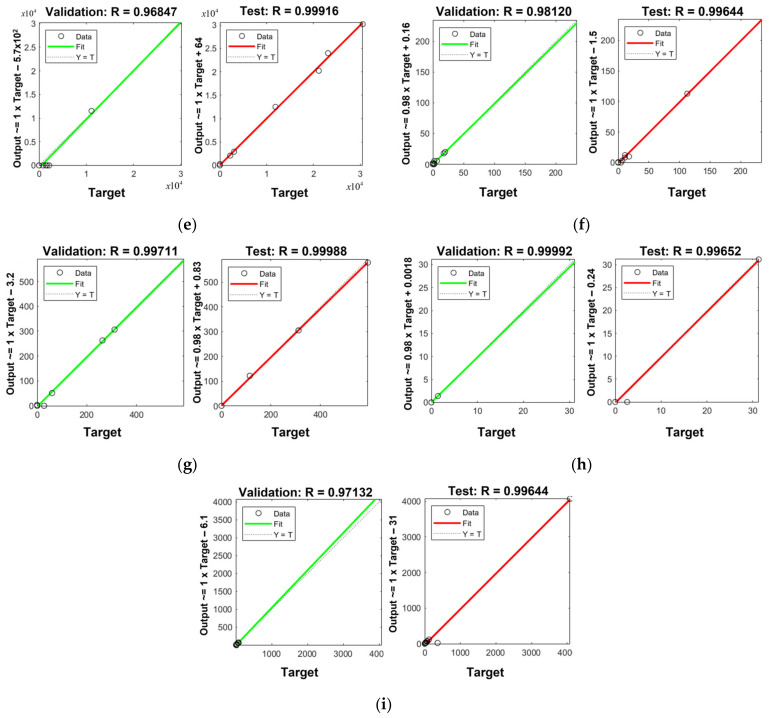
Regression plots for soils: (**a**) Arsenic, (**b**) Barium, (**c**) Chromium, (**d**) Copper, (**e**) Iron, (**f**) Lead, (**g**) Manganese, (**h**) Nickel, (**i**) Zinc.

## Data Availability

All data are contained in the manuscript.
